# Annual assessment of the wastewater treatment capacity of the microalga *Scenedesmus almeriensis* and optimisation of operational conditions

**DOI:** 10.1038/s41598-021-01163-z

**Published:** 2021-11-04

**Authors:** Ana Sánchez-Zurano, Ainoa Morillas-España, Cintia Gómez-Serrano, Martina Ciardi, Gabriel Acién, Tomás Lafarga

**Affiliations:** 1 Department of Chemical Engineering, University of Almería, 04120 Almería, Spain; 2 CIESOL Solar Energy Research Centre, Joint Centre University of Almería-CIEMAT, 04120 Almería, Spain

**Keywords:** Chemical engineering, Biotechnology, Environmental sciences

## Abstract

The depth of the culture and the dilution rate have a striking effect on the biomass productivity and the nutrient recovery capacity of microalgal cultures. The combination of culture depth and dilution rate that allows to maximise the performance of the system depends on environmental conditions. In the current study, a response surface methodology was used to explore the relationship between the two most relevant operational conditions and the biomass productivity achieved in 8.3 m^2^ pilot-scale raceways operated using urban wastewater. Four polynomial models were developed, one for each season of the year. The software predicted biomass productivities of 12.3, 25.6, 32.7, and 18.9 g·m^−2^·day^−1^ in winter, spring, summer, and autumn, respectively. The models were further validated at pilot-scale with *R*^2^ values ranging within 0.81 and 0.91, depending on the season. Lower culture depths had the advantage of minimising nitrification and stripping but allow to process a lower volume of wastewater per surface area. Biomass productivity was higher at culture depths of 0.05 m, when compared to 0.12 and 0.20 m, while the optimal dilution rate was season-dependent. Results reported herein are useful for optimising the biomass productivity of raceway reactors located outdoors throughout the year.

## Introduction

The overexploitation of water is a serious environmental concern. The discharge of effluents that contain heavy metals and an excessive amount of nutrients (namely nitrogen and phosphorus) lead to eutrophication and pollution of water reservoirs^1^. Today, approximately 850 M people in the world lack access to safe water and it is estimated that 1.8 M people die annually due to diseases linked to consumption of contaminated water^2^. The situation is critical and for this reason ensuring sufficient and safe water supplies for everyone has become a central aspect of the 2030 Agenda and on Sustainable Development Goals of the United Nations^3^.

The high content of nitrogen and phosphorus turns contaminated water an optimal culture medium for the growth of photo-autotrophic organisms such as microalgae^4^. Microalgae have the capacity of efficiently recovering nutrients from wastewater and other wastes while simultaneously producing not only clean water but also valuable biomass with unlimited possibilities^5–7^. Microalgal production with wastewater has been traditionally carried out in open ponds or raceways. The biomass production capacity of open systems such as raceway reactors is limited by several variables that determine the success of the process. These include abiotic factors (light availability, temperature, pH, dissolved oxygen concentration, and availability of nutrients nutrients) and process-related factors such as the design of the photobioreactor, mixing, culture depth, and amount of biomass daily harvested^4,8^. Improving the productivity of microalgae photobioreactors has become the goal of most of the research groups and companies working with microalgae^9^. Several studies have been carried assessing the effect of environmental factors on microalgae production and multiple mathematical models have been developed considering these variables^10–13^. However, the number of studies comparing the annual productivity of open photobioreactors at pilot- or large-scale is very limited^14^. Moreover, little is known on how operational conditions affect microalgae production. The two most relevant operational parameters of raceway reactors are the cultures’ depth and the dilution rate (or the hydraulic retention time). Traditionally, open raceways for wastewater treatment were operated at water depths ranging from 0.2 to 0.4 m and hydraulic retention times of between 7 and 10 days. Under these conditions, light availability for the microalgal cells is inadequate because of the self-shading effect of microalgae, and therefore microalgal growth and productivity are limited. Operating at high water depths involves that the nitrogen supply is higher than the microalgal recovery capacity giving rise to an overload of the system and the growth of nitrifiers^4^. For these reasons, novel reactor designs such as thin-layer cascade reactors with culture depths lower than 2 cm have been developed. These systems allow a greater light availability promoting microalgal growth and high biomass concentrations^15^. The main drawback of these reactors, when operated using wastewater, is that the amount of wastewater that can be processed per surface area is lower^16^.

The aim of the current study was to optimize the annual production of the microalga *Scenedesmus almeriensis* using urban primary wastewater and a response surface methodology (RSM). The goal was to identify the optimal operational parameters, namely culture depth and dilution rate, for each season. Experiments were conducted outdoors using pilot-scale raceway reactors operated in semi-continuous mode. Four polynomial models were developed, one for each season, and the optimal combinations of depth of the culture and dilution rate were identifying. Up to the best of the authors’ knowledge, this is the first time that a wastewater treatment process is modelled using a RSM and data generated in pilot-scale conditions.

## Results and discussion

### Biomass productivity

**Figure 1:**
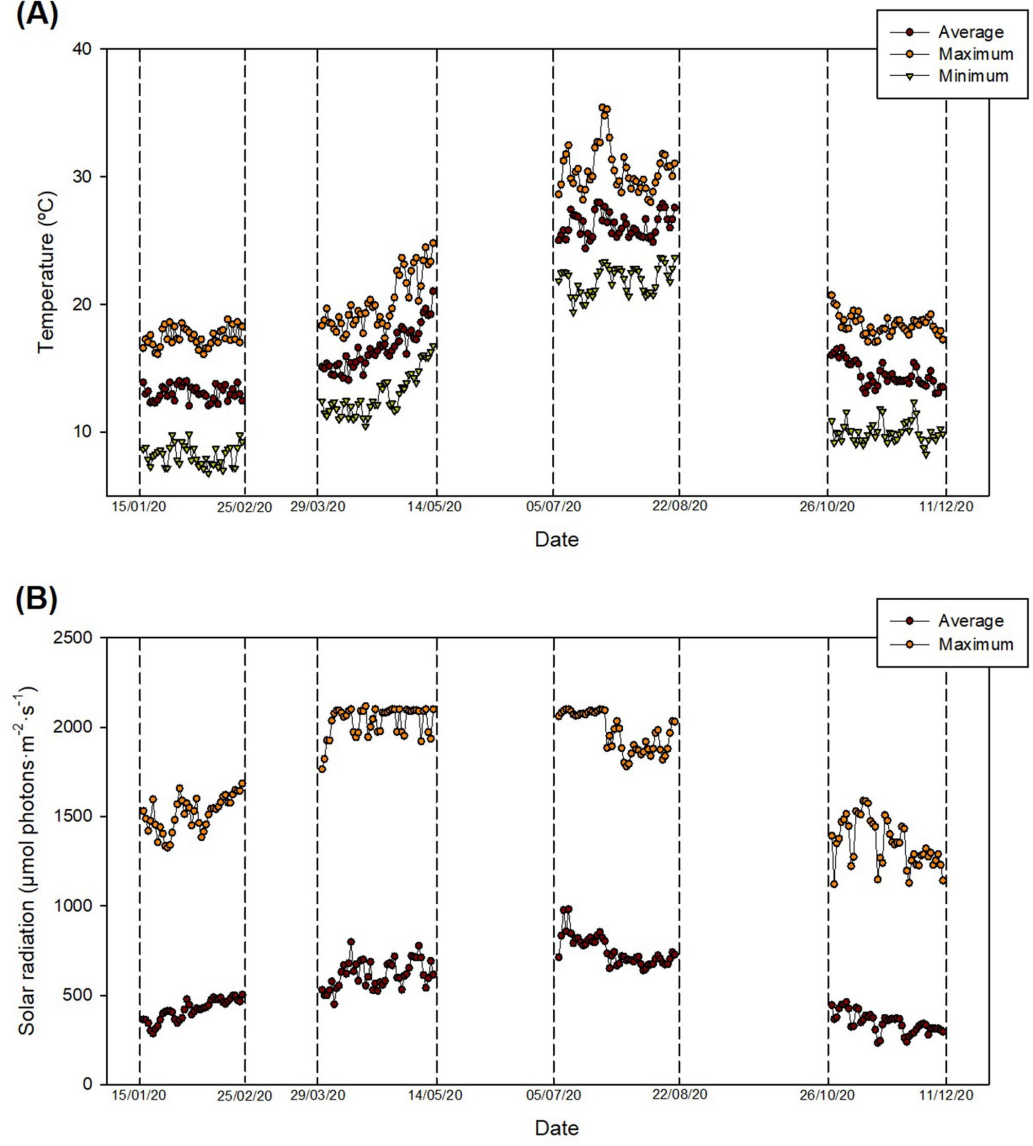
**(A)** Temperature and **(B)** solar radiation during the experimental runs. Determinations were conducted every 1 s. Average values represent the average of all the measurement taken in one day. Maximum and minimum values represent the maximum and minimum temperature or solar radiation value determined per day. Figure made using SigmaPlot v11.0 (Systat Software Inc., CA, US).

Biomass productivity values were highly influenced by environmental conditions, with higher values observed in spring and summer when compared to autumn and especially winter (Table 1). Light availability is the most important factor influencing microalgae production and therefore this variability was expected. Environmental conditions during the experimental periods are shown in Fig. 1. The average temperature was 13.1 ± 0.6, 16.5 ± 1.2, 26.2 ± 1.0, and 14.5 ± 0.9 ºC in winter, spring, summer, and autumn, respectively. Because of the mild winter season of the region, average winter temperatures were comparable to those of autumn. The main difference between winter and autumn was the average minimum temperature, which was 8.1 ± 0.6 ºC in the former and 10.6 ± 0.8 ºC in the latter. The number of sunlight hours as well as the solar radiation intensity also changes between the different seasons. Average irradiation values (considering only the illuminated period) of 418 ± 58, 618 ± 76, 748 ± 73, and 386 ± 57 µmol photons·m^-2^·day^-1^ in winter, spring, summer, and autumn, respectively. The main difference between winter and autumn was the number of sunlight hours being 10.4 h on average in winter and 11.0 h in autumn. Approximately 13.1 and 14.5 h of solar radiation values were recorded in spring and summer, respectively.

**Table 1:** Central composite response surface designs for biomass productivity in winter (DM1), spring (DM2), summer (DM3) and autumn (DM4).

	Coded variables		Actual variables: operational parameters		Response: biomass productivity (P_b_)			
Run^a^	A: Dilution rate	B: Depth	A: Dilution rate (day^−1^, D)	B: Depth (m, h)	DM1^b^: (g·m^−2^·day^−1^)	DM2^b^: (g·m^−2^·day^−1^)	DM3^b^: (g·m^−2^·day^−1^)	DM4^b^: (g·m^−2^·day^−1^)
1	0	0	0.32	0.13	12.30	20.1	23.9	15.8
2	0	0	0.32	0.13	11.11	22.7	27.04	16.1
3	0	0	0.32	0.13	10.32	21.5	22.9	16.7
4	−1	1	0.15	0.20	8.50	12.4	10.8	10.6
5	−1	0	0.15	0.13	10.91	13.3	16.9	11.5
6	−1	−1	0.15	0.05	11.5	15.4	15.9	15.7
7	1	−1	0.50	0.05	8.5	24.6	32.4	13.9
8	0	1	0.33	0.20	9.2	17.2	19.3	12.9
9	1	0	0.50	0.13	8.2	20.9	28.5	11.0
10	0	-1	0.33	0.05	11.8	26.3	30.2	19.6
11	1	1	0.50	0.20	7.0	15.3	17.2	9.1

^a^Run number does not correspond to the number of processing, which was conducted randomly.

^b^Values represent the mean of two independent experiments. DM1, DM2, DM3, and DM4 were the set of experiments conducted in winter, spring, summer, and autumn, respectively.

**Figure 2:**
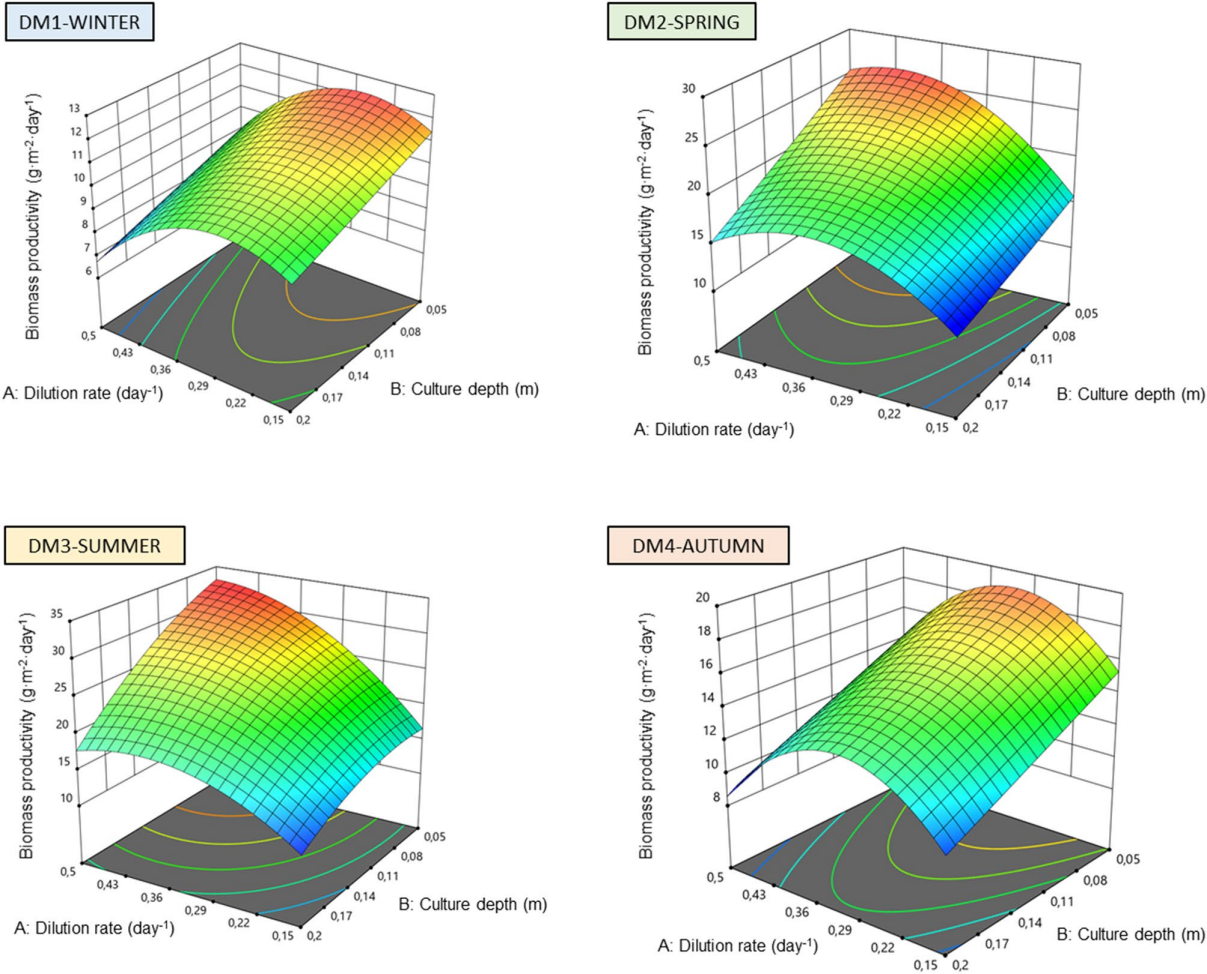
Effect of dilution rate and culture depth on biomass productivity during winter (DM1), spring (DM2), summer (DM3) and autumn (DM4). Figure made using Design Expert v11.0 (Stat-Ease Inc., MN, US).

**Figure 3:**
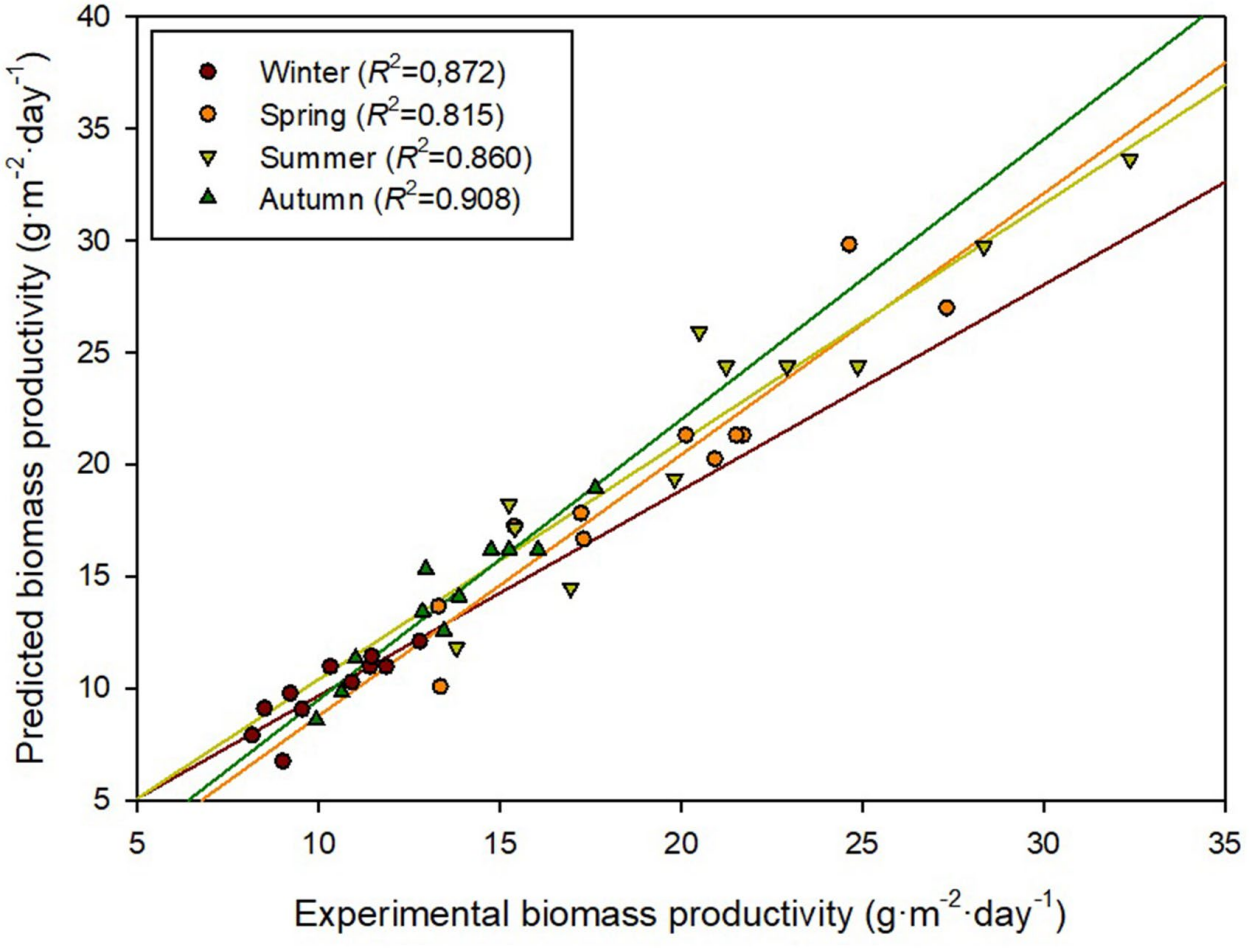
Scatter plot of predicted and experimental biomass productivity values. Figure made using SigmaPlot v11.0 (Systat Software Inc., CA, US).

Because of the high dependence of microalgal growth on environmental conditions, namely solar radiation and temperature, four different models were developed: DM1, winter; DM2, spring; DM3, summer; and DM4, autumn. These designs generated 11 experimental runs, listed in Table 1. Data from the different experimental runs, biomass productivity values, could be fitted by polynomial quadratic equation in term of the studied variables: the cultures’ depth and the dilution rate used. The application of RSM led to the following quadratic equations in terms of actual factors:

- Winter, DM1:
  $${P}_{b}\left(\mathrm{g}\cdot {\mathrm{m}}^{-2}\cdot {\mathrm{day}}^{-1}\right)=8.68+32.60\cdot A-15.53\cdot B-60.56\cdot {A}^{2}$$
- Spring, DM2:
  $${P}_{b}\left(\mathrm{g}\cdot {\mathrm{m}}^{-2}\cdot {\mathrm{day}}^{-1}\right)=5.90+113.41\cdot A-47.82\cdot B-145.43\cdot {A}^{2}$$
- Summer, DM3:
  $${P}_{b}\left(\mathrm{g}\cdot {\mathrm{m}}^{-2}\cdot {\mathrm{day}}^{-1}\right)=-0.23+149.57\cdot A-6.73\cdot B-192.00\cdot A\cdot B-142.74\cdot {A}^{2}$$
- Autumn, DM4:
  $${P}_{b}\left(\mathrm{g}\cdot {\mathrm{m}}^{-2}\cdot {\mathrm{day}}^{-1}\right)=7.33+86.23\cdot A-36.73\cdot B-138.11\cdot {A}^{2}$$
  where ${P}_{b}$ is the predicted biomass productivity and $A$ and $B$ are the dilution rate (day^-1^) and the depth of the culture (m), respectively. These equations represent empirical relationships between the biomass productivities that could be obtained during each season and the independent variables. It is important to highlight that those variables and interactions between variables that were not significant to the model were deleted and not considered. Figure 2 represents the 3D contour model graphs generated by Design Expert v.11 showing the combinations of culture depths and dilution rates that lead to higher biomass productivities. The complete statistical analysis of the proposed quadratic models is provided as Supplementary Material 1. Briefly, the analysis revealed that DM1–4 were adequate (*p* = 0.0018, DM1; *p* < 0.0001, DM2; *p* = 0.0008 DM3, and *p* < 0.0001 DM4) with good determination coefficients (*R*^2^ = 0.869, DM1; *R*^2^ = 0.972, DM2; *R*^2^ = 0.941, DM3; *R*^2^ = 0.863, DM4). The predicted and adjusted *R*^2^ values were in reasonable agreement, with a difference lower than 0.2, and the lack of fit *F*-values were not significant relative to the pure errors, confirming the validity of the models. In addition, a no significant lack of fit was obtained in the four models which suggests that they properly fit the prediction across the designed space, which means at culture depths within 0.05–0.20 m and dilution rates of 0.15–0.50 day^−1^.
  Culture depth and dilution rate affected biomass productivity during the four seasons (*p* < 0.001) as well as the combination of both factors in spring and summer (*p* < 0.05). It is likely that the higher temperature and solar radiation values that reached the cultures during spring and summer had a higher influence on the productivity of the reactors and therefore, the combination of both factors was also significant. Perturbation plots are provided as Supplementary Material 2. Biomass productivity was more sensitive to the dilution rate imposed than to the depth of the culture, although both factors were significant. Maximum biomass productivity values obtained in winter, spring, summer, and autumn were 12.3, 26.3, 32.4, and 19.6 g·m^−2^·day^−1^, respectively. These values were higher than those reported in previous reports using raceway reactors and the same microalga, with an average annual biomass productivity of approximately 17 g·m^−2^·day^−1^ ^15^. This difference can be attributed to the smaller scale of the reactor used in the current study, which facilitates mass transfer and the control of the process, and the optimisation of the operational conditions especially the cultures depth. Indeed, the highest biomass productivity was achieved when operating at a depth of 0.05 m and a dilution rate of 0.5 day^−1^. The low depth of the culture allows a higher light availability as it minimises the self-shading effect of microalgae. This is the main reason why thin-layer cascade reactors, with culture depths of 0.5–5.0 cm are so productive when compared to raceways^17^. The highest productivity obtained at the highest dilution rate in summer can be attributed to a higher nutrient and light availability. A larger amount of culture was replaced with fresh medium every day, reducing biomass concentration (less shading) and increasing the amount of nutrients introduced into the system. Varying the dilution rate can also be used as a strategy to control the temperature of microalgal cultures^18^ and this could have also partially contributed to the observed biomass productivities.
  In order to ensure the quality and reliability of DM1-4, the four models were validated using the same procedures followed to generate the model. Data, shown in Fig. 3 demonstrated that the predicted and experimental biomass productivity values were in good agreement with correlation coefficients of 0.8–0.9 depending on the season. After the validation of the models, the designed models DM1–4 were optimised using Design Expert v.11. The software predicted the optimum combination of culture depth and dilution rate for each season. The set goal was to maximise biomass productivity while keeping the dilution rate and the depth of the culture within the limits of the model (0.15–0.50 day^−1^ and 0.05–0.20 m, respectively). The software predicted the following combinations as the optimal:
- Winter, DM1: A maximum biomass productivity of 12.3 g·m^−2^·day^−1^ was predicted when operating the reactors at a dilution rate of 0.27 day^−1^ and a depth of the culture of 0.05 m (Desirability = 0.99).
- Spring, DM2: A maximum biomass productivity of 25.6 g·m^−2^·day^−1^ was predicted when operating the reactors at a dilution rate of 0.39 day^−1^ and a depth of the culture of 0.05 m (Desirability = 0.95).
- Summer, DM3: A maximum biomass productivity of 32.7 g·m^−2^·day^−1^ was predicted when operating the reactors at a dilution rate of 0.49 day^−1^ and a depth of the culture of 0.06 m (Desirability = 1.00).
- Autumn, DM4: A maximum biomass productivity of 18.9 g·m^−2^·day^−1^ was predicted when operating the reactors at a dilution rate of 0.31 day^−1^ and a depth of the culture of 0.05 m (Desirability = 0.94).

These conditions will need to be further validated in future studies. Results suggested that it is important to select the optimum dilution rate for each season of the year to maximise biomass productivity and that lower culture depths promote biomass production by increasing light availability. This supports previous findings that demonstrated the higher productivity of reactors with lower depths^14–16,19,20^. Finally, it is important to highlight that the produced biomass was not *Scenedemus almeriensis* alone but a combination of different eukaryotic and prokaryotic microorganisms. In the current study, the dominant strain of the consortia was *Scenedesmus* sp. identified by microscopic examination. Previous reports demonstrated the capacity of *Scenedesmus* strains to be the dominant eukaryote in open reactors in the south of Spain independently of the operational conditions—although the composition of the microalgae-bacteria consortia is affected by both environmental and operational conditions^14,21^.

### Nutrient removal capacity

Producing high quantities of microalgal biomass is important to render microalgae production economically viable. The use of wastewater as the nutrient source was initially suggested as a strategy to reduce production costs. However, because of the need for reclaiming used water (and disposing safe water) and the high capacity of microalgae to recover nutrients from waste, nowadays, microalgal biomass can be considered as a co-product of wastewater treatment processes. A valuable co-product as it can be utilised in varied industries including agriculture, feed and aquafeed production, and production of biogas, among other applications^22,23^.

**Figure 4:**
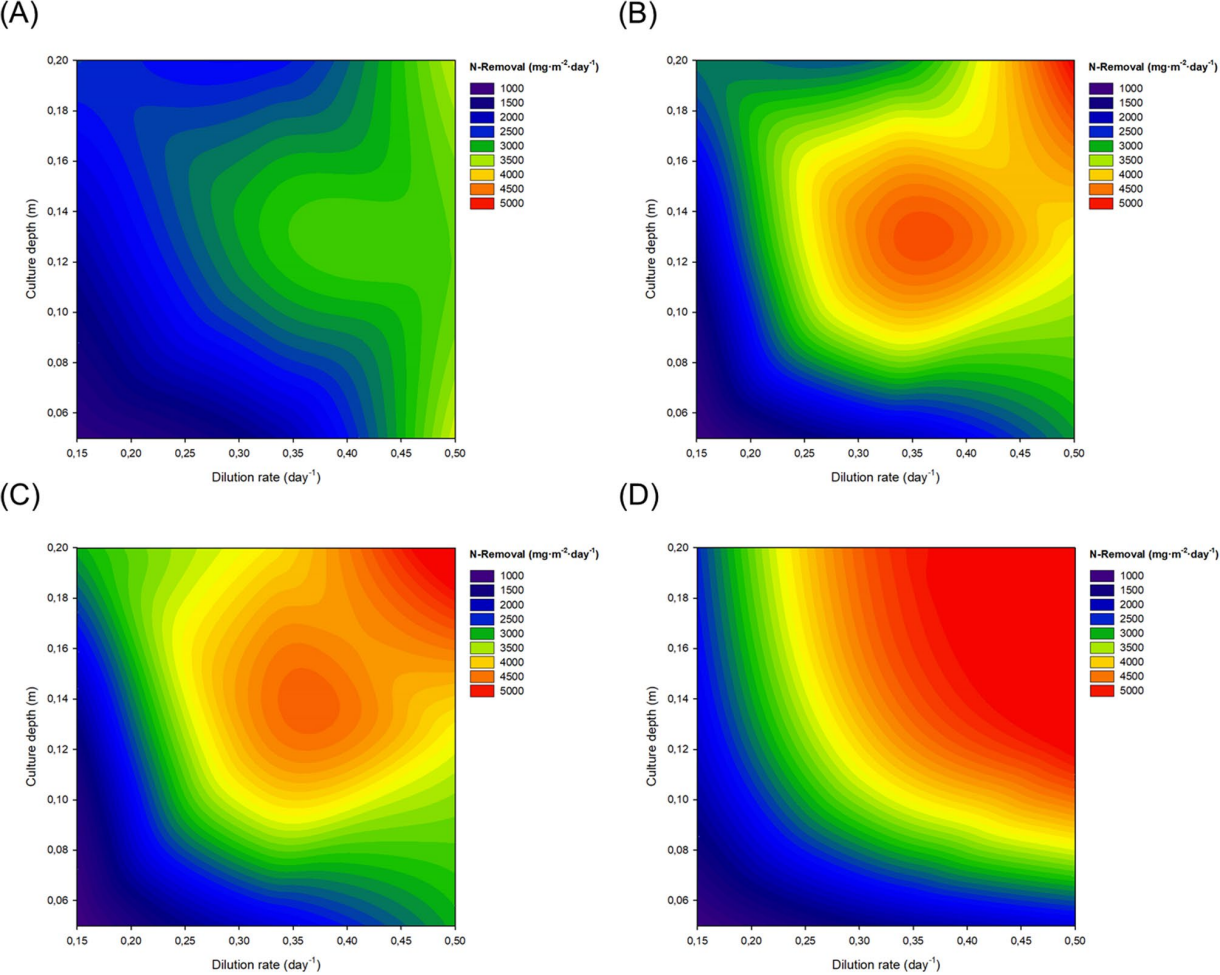
2D contour plot of total nitrogen (N-NH_4_^+^ and N-NO_3_^−^) removal capacity of the reactors in **(A)** winter, **(B)** spring, **(C)** summer, and **(D)** autumn. Figure made using SigmaPlot v11.0 (Systat Software Inc., CA, US).

Anthropogenic inputs of nitrogen and phosphorus are the main causes of eutrophication^24^. In the current study, the content of N-NH_4_^+^ and N-NO_3_^−^ in the inlet and outlet effluents of the reactors was determined (Supplementary Material 3). Overall, the main inorganic nitrogen source was ammonia, with N-NH_4_^+^ concentrations in the inlets that varied from 55 to145 mg·L^−1^. The content of N-NO_3_^-^ in the inlets was much lower with values ranging within 2–11 mg·L^−1^. The total nitrogen removal capacity of the system is showed in Fig. 4. It is important to highlight that in the current study, total nitrogen refers to the sum of N-NH_4_^+^ and N-NO_3_^−^ but there are other nitrogen species (nitrites, urea, protein, etc.) that contributed to the real total nitrogen content of the wastewater that were not considered. Therefore, results presented in the current study represent an estimation of the total nitrogen removal capacity of the reactors and describe N-NH_4_^+^ and N-NO_3_^−^ recovery capacities. Overall, a higher nitrogen removal capacity was observed when operating the reactors at higher dilution rates and higher culture depths. This was mainly caused by a larger amount of water being processed per surface area when higher dilution rates and larger culture depths were used. The N-NH_4_^+^ removal capacity was close to 100%, with average values of 97.2, 96.9, 98.7, and 98.8% in winter, spring, summer, and autumn independently of the operational conditions used. The content of N-NH_4_^+^ and N-NO_3_^−^ in the outlets was (for most of the studied experimental runs) below the maximum discharge limits of Spanish regulations, set at 10–15 mg·L^−1^ of nitrogen^25^. However, in some runs the concentration of nitrogen exceeded this maximum threshold, especially when inlet concentration of nitrogen was higher than 100 mg·L^−1^. The current study aimed at maximising biomass productivity. However, when wastewater with a high nitrogen content is used it is likely that the wastewater will need to be pre-diluted or a tertiary treatment should be introduced into the process to ensure a safe disposal of the outlet effluents. Reducing the dilution rate could also be used as a strategy to reduce the nitrogen content in the outlets. Further studies will determine the potential of *S. almeriensis* to remove organic nitrogen, which was not considered in the current study. Not all the N-NH_4_^+^ removed from the water was used for microalgal growth. Part of this nitrogen was converted by nitrifying bacteria intro nitrates, which is a common reaction taking place in wastewater treatment processes^26^. This is clear from the data as the content of nitrates in the outlets of the reactors was higher than in the inlets in most experimental runs, especially when operating the reactors at higher culture depths (Supplementary Material 3). This phenomenon was observed previously and it can be attributed to the higher light availability in cultures being produced with lower culture depths, which promotes phototrophic growth^14^. Microalgae can use N-NO_3_^−^ as the source of nitrogen. Indeed, at industrial level, many microalgae producers use NaNO_3_ as the nitrogen source. However, based on the higher N-NO_3_^−^ content in the outlets than in the inlets it is likely that the studied strain used N-NH_4_^+^ as the main nitrogen source. This suggestion is in line with previous reports that suggested that in the presence of both, N-NH_4_^+^ and N-NO_3_^−^, the majority of microalgal strains prefer the former^27^.

Assuming a nitrogen content of the produced biomass of 10%, a mass balance conducted to the reactors suggested that part of the N-NH_4_^+^ removed from the wastewater was desorbed into the atmosphere. This means that the ammonium ion that is dissolved into the water transits to ammonia gas that is liberated into the atmosphere and therefore, the high nitrogen removal rates from the water do not correspond to high nitrogen recoveries as it was highlighted in previous reports^28^. Because of the varying ammonia concentration of the wastewater used, and the limitations of the current study that did not assess nitrites and organic nitrogen, it is not possible to identify how operational conditions affected ammonia stripping. High pH values significantly affect the ammonia/ammonium ratio in favour of ammonia. This is the main factor affecting ammonia stripping. The consumption of inorganic carbon by autotrophic organisms leads to an increase in the pH of the culture. However, it was not the case in the current study as the pH was controlled by on-demand injection of carbon dioxide. High temperatures can also promote the loss of nitrogen via stripping. Aeration and agitation are other key factors affecting stripping^29^. Further studies will optimise the aeration and mixing of the reactors to minimise the loss of nutrients while maintaining a correct agitation to avoid sedimentation.

**Figure 5:**
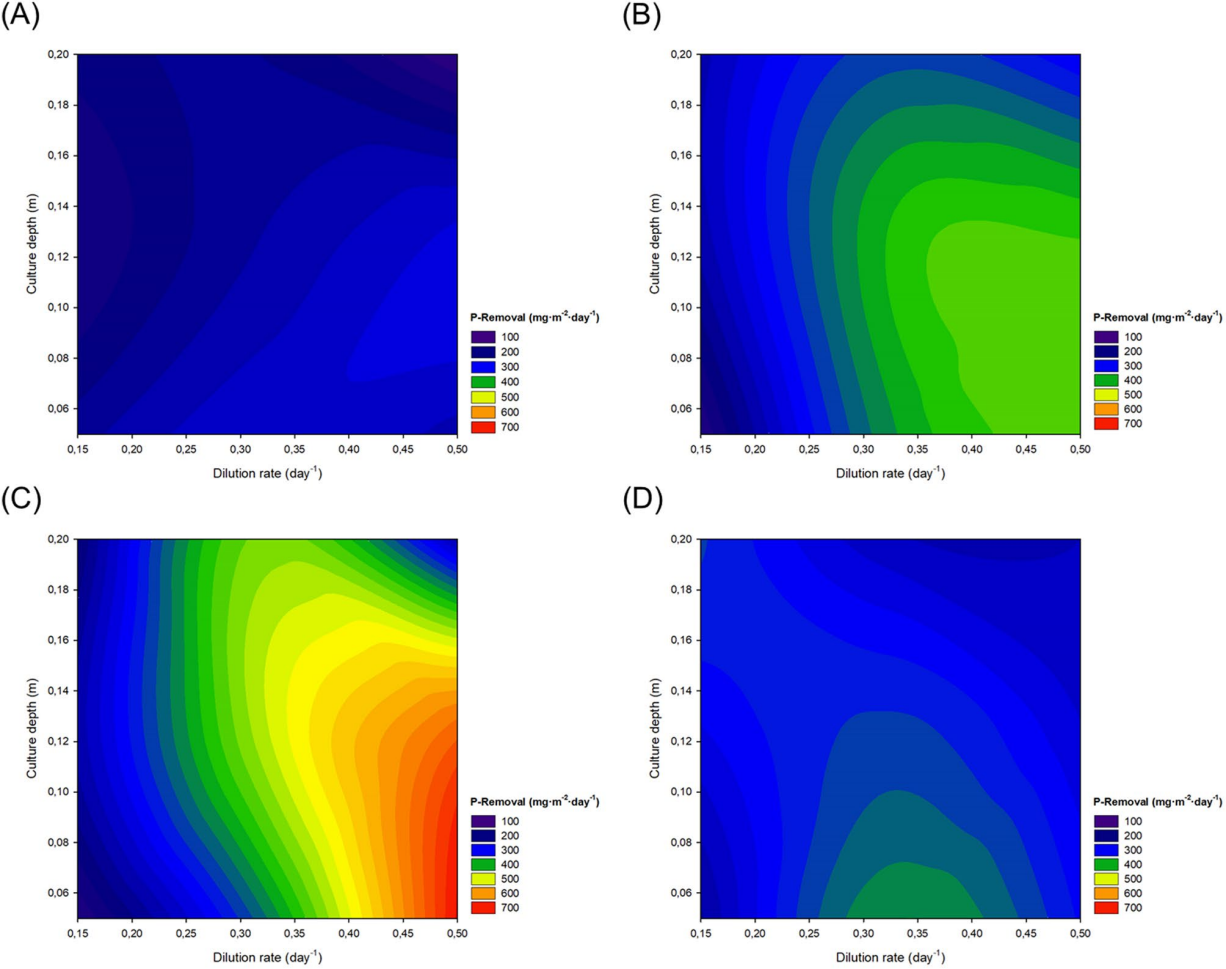
2D contour plot of P-PO_4_^3−^ removal capacity of the reactors in **(A)** winter, **(B)** spring, **(C)** summer, and **(D)** autumn**.** Figure made using SigmaPlot v11.0 (Systat Software Inc., CA, US).

The capacity of the system to recover P-PO_4_^3−^ is shown in Fig. 5. Values were lower than those obtained for nitrogen mainly because of the lower content of the inlets (3–21 mg·L^−1^) and the lower phosphorus requirements of microalgal biomass. P-PO_4_^3−^ removal values were in line with those obtained for biomass productivity, demonstrating that the P-PO_4_^3−^ removed from the water was recovered to produce biomass. Higher P-PO_4_^3−^ removal values were achieved when operating the reactors at higher dilution rates and lower depths. Higher dilution rates allow to process larger volumes of water and therefore more nutrients are introduced into the reactor increasing nutrient availability. Moreover, by diluting the culture light availability is also increased. As highlighted before, lower culture depths promote microalgal biomass production and these are the main reasons why higher P-PO_4_^3−^ removal values were achieved when operating at lower culture depths and higher dilution rates. However, it is important to highlight that the optimal conditions should be assessed independently for each season, as biomass productivity is highly influenced by environmental conditions. Indeed, in the current study, the productivity achieved when operating at 0.05 m of culture depth and 0.50 day^−1^ was 3.8-fold higher in summer than in winter. The P-PO_4_^3−^ concentration in the different wastewaters used varied significantly as well as the concentrations in the outlets. When the P-PO_4_^3−^ concentration was lower than 9–10 mg·L^−1^, the process was able to meet the maximum discharge level of Spanish regulations set at 1–2 mg·L^−1^^25^. However, as it happened with total nitrogen, the system was not able to meet this maximum discharge value when the inlet P-PO_4_^3−^ concentration was higher than 10 mg·L^−1^, suggesting that it is likely that the dilution rate should be reduced or the wastewater diluted when high P-PO_4_^3−^ concentrations are detected. It is important to highlight that the current study aimed at optimising biomass productivity.

Overall, environmental and operational conditions had a striking effect on microalgal growth and biomass productivity. Operating at higher culture depths allowed higher nitrogen removals from primary wastewater, although nitrification and stripping were also promoted and not all the nitrogen was used for biomass production. Lower culture depths have the advantage of minimising nitrification and stripping but allow to process a lower volume of wastewater per surface area. As phosphorus is not desorbed into the atmosphere, the phosphorus removal capacity of the system was improved when biomass productivity was higher. Therefore, the goal should be to maximise biomass productivity. Because of (inevitably) changing environmental conditions, the optimum operational conditions should be assessed for each month/season and location. This demonstrates the importance of designing photobioreactors that can be easily adjusted to provide the microalgae-bacteria consortia with the optimal conditions. Statistical approaches such as response surface methodology can be used to maximise microalgae biomass production by optimising operational parameters. It is important to consider the effect of environmental conditions on biomass productivity. In the current study, lower culture depths favoured microalgal growth in all the experimental periods assessed. However, the dilution rate is highly dependent of solar radiation and temperature being the optimal higher in those months with higher photosynthetic activity. Further studies will up-scale biomass production using the identified optimal parameters for each season and assess the effect of operational parameters not only on biomass productivity but also on the quality of the produced biomass, as it is known that environmental and operational parameters can promote the production of valuable biomolecules.

## Methods

### Biomass production

#### Selected strains

The strain selected was *Scenedesmus almeriensis* CCAP 276/24 available at the Department of Chemical Engineering of the University of Almería (Spain). This strain was isolated for the first time inside a greenhouse in the Almería and therefore is well adapted to the environmental conditions of the regions. The initial inocula were prepared using 5 L controlled photobioreactors maintained at 23 ºC, pH 8.0, and 500 µmol photons·m^−2^·s^−1^. Once the biomass concentration reached 1.0–1.5 g·L^−1^, the culture was up-scaled using 80 L pH-controlled bubble columns located at the pilot-plant facilities of the University of Almería in the Andalusian Institute of Agricultural and Fisheries Research and Training (Almería, Spain). The culture medium used to produce the inocula was made using commercial fertilisers instead of pure chemicals: 0.90 g·L^−1^ NaNO_3_, 0.18 g·L^−1^ MgSO_4_, 0.14 g·L^−1^ K_2_PO_4_, and 0.03 g·L^−1^ of Karentol (Kenogard, Spain). The latter is a commercial mixture of micronutrients including boron, copper, iron, manganese, and zinc.

#### Culture medium composition

Pilot-scale production was carried out using urban primary wastewater as the sole nutrient source. Wastewater was collected from an urban wastewater treatment plant in Almería (Spain). The composition of the wastewater varied significantly between the different experiments (Supplementary Material 3). Inorganic nitrogen species determined were N-NH_4_^+^ and N-NO_3_^−^, which varied between 55 and 145 and 2–11 mg·L^−1^, respectively. In the current study, total nitrogen refers to the sum of both inorganic species, being N-NH_4_^+^ the most relevant inorganic nitrogen source. Nitrites and organic nitrogen sources were not considered but also contributed to the availability of nitrogen in the cultures. P-PO_4_^3−^ of the wastewater effluents varied between 3 and 21 mg·L^−1^ and the chemical oxygen demand (COD) of the inlets was 690–890 mg·L^−1^ (Supplementary Material 3).

#### Photobioreactors used and operational conditions

Microalgal biomass was produced using three identical raceway photobioreactors located outdoors which were considered the experimental units. The reactors were inoculated with 10% of their total volume using *S. almeriensis* cultures produced using bubble columns as described above (the biomass concentration of the inocula was approximately 1.0 g·L^−1^). The reactors were built of polypropylene and consisted of two 6 m long and 0.6 m wide channels connected by 180º curves (8.33 m^2^). The reactors were lifted from the ground approximately 1 m and included a 170 L collector where air was injected at 15 L·min^-1^ to improve mass transfer and avoid an excessive oxygen accumulation. The reactors were operated in semi-continuous mode at dilution rates varying within 0.15–0.50 day^−1^ (Table 1). This means that every day 15–50% (depending on the experimental run) of the total cultures volume was harvested and replaced with primary wastewater. The depth of the cultures varied within 0.05 to 0.20 m. Therefore, the volume of the culture varied from 586 to 1840 L, depending on the cultures’ depth. All three reactors were continuously online monitored and controlled using a supervisory control and data acquisition (SCADA) system. Ambient temperature and solar radiation values were recorded every 1 s during the different experimental runs. Data is available at http://sabana.ual.es/. The average, maximum, and minimum values are summarised in Fig. 1. The pH of the culture was controlled at 8.0 by on-demand injection of carbon dioxide. The reactors were operated until the total volume of the reactor was replaced at least twice and the biomass concentration was constant for at least three consecutive days. The outlet of the reactors at the pseudo steady-state (3 last days) was collected and either analysed immediately or stored at −20 ºC until further analysis. Once each experimental run was finished, the reactors were cleaned and re-inoculated as described above.

### Experimental design

A response surface methodology was carried out to optimise biomass productivity by modifying the most relevant and easy to adjust operational conditions. Because of the high dependence of productivity on environmental conditions, four separate experiments were designed using Design Expert v.11 (Stat-Ease Inc., MN, USA). These were termed as DM1, DM2, DM3, and DM4 and were conducted during winter, spring, summer, and autumn, respectively (Fig. 1).

DM1-4 investigated the effect of two independent variable, namely culture depth and dilution rate, on biomass productivity. Using a central composite face-centred design, the software generated 11 combinations listed in Table 1—the central points were repeated three times to assess the error within the model. The culture depth varied between 0.05 and 0.20 m and the dilution rate varied from 0.15 to 0.50 m^−1^. Biomass concentration and nutrient content were determined in triplicate and the average was used as the response. Experimental data were fitted to a polynomial response surface (best fit), predicted by the following equation:

$$Y={\beta }_{0}+\sum_{i=1}^{n}{\beta }_{i}{X}_{i}+\sum_{i=1}^{n}{\beta }_{ii}{{X}_{i}}^{2}+\sum_{i=1}^{n}\sum_{j=i+1}^{n}{\beta }_{ij}{X}_{i}{X}_{j}$$

where $Y$ is the dependent variable, ${\beta }_{0}$ is the centre point of the system, ${\beta }_{i}$, ${\beta }_{ii}$, and ${\beta }_{ij}$ are the coefficients of the linear, quadratic, and interactive effect, and ${X}_{i}$,${{X}_{i}}^{2}$, and ${X}_{i}{X}_{j}$ are the linear, quadratic, and interactive effect of the independent variables. Non-significant terms (*p* < 0.05) were deleted from the polynomial model after ANOVA analysis and a new ANOVA was performed to obtain the coefficients of the final equation. All the models were further validated and optimisation was done as described previously^30^.

### Analytical determinations

Biomass concentration was determined gravimetrically after filtration and oven-drying at 80 ºC during 24 h. Biomass productivity was calculated as the product of biomass concentration and the dilution rate (0.15–0.50 day^−1^). The maximum quantum yield of photosystem II chemistry was measured after 10 min of adaptation to the dark using an AquaPen AP 100 fluorometer (Photon System Instruments, Czech Republic). The concentration of N-NH_4_^+^, N-NO_3_^−^, and P-PO_4_^3-^ was determined spectrophotometrically using standard official methods approved by the Spanish Ministry of Agriculture^31^. The P-PO_4_^3−^ content was determined spectrophotometrically through the phospho-vanado-molybdate complex using a Genesys 10S spectrophotometer (Thermo Fisher Scientific, Spain). N-NO_3_^-^ was quantified at 220–275 nm using a GENESYS 10S UV–Vis spectrophotometer (Thermo Fisher Scientific, Spain) and N-NH_4_^+^ was measured using the Nessler reactive method. The COD of the wastewater was determined using LCI-400 commercial kits (Hach Company, UK).

## Supplementary Information

Supplementary Information 1.

Supplementary Information 2.

Supplementary Information 3.
