# Supplementary figures and images for: Annual assessment of the wastewater treatment capacity of the microalga Scenedesmus almeriensis and optimisation of operational conditions
Source: Sci Rep. 2021 Nov 4;11:21651. doi: 10.1038/s41598-021-01163-z (PMC8569198; doi:10.1038/s41598-021-01163-z)

**Supplementary Material 1. Statistical analysis of the proposed DM1-4 models**

**
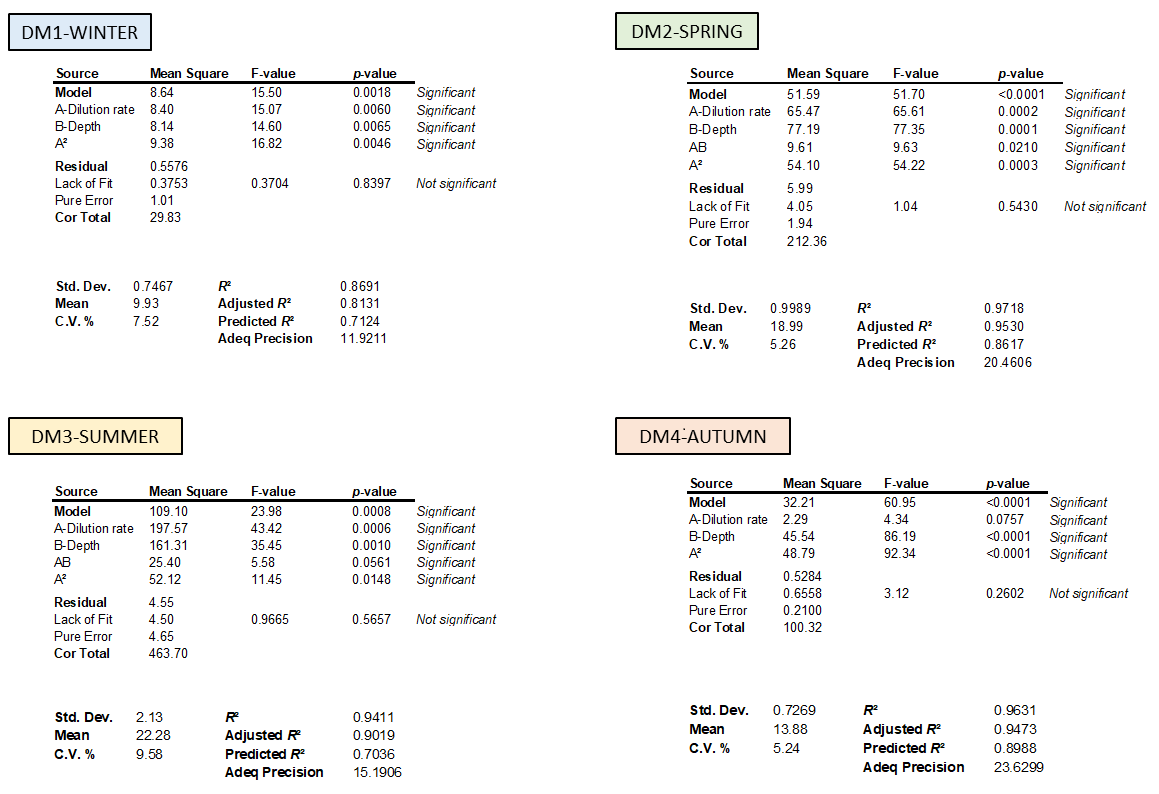
**

Supplement: Supplementary file 1 — Supplementary Information 1. [file 41598_2021_1163_MOESM1_ESM.docx]

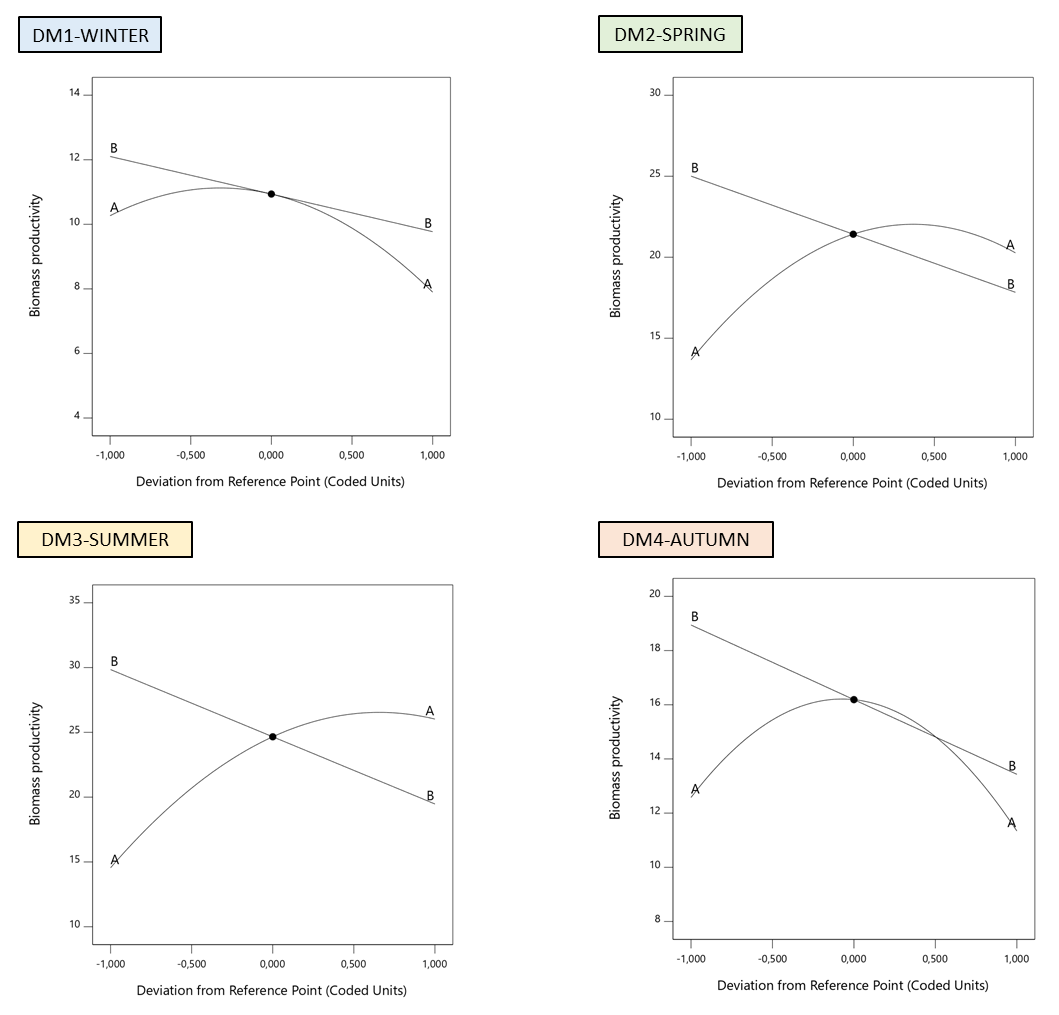
**Supplementary Material 2. Perturbation plots**

Supplement: Supplementary file 2 — Supplementary Information 2. [file 41598_2021_1163_MOESM2_ESM.docx]
