# Supplementary material for: Annual assessment of the wastewater treatment capacity of the microalga Scenedesmus almeriensis and optimisation of operational conditions
Source: Sci Rep. 2021 Nov 4;11:21651. doi: 10.1038/s41598-021-01163-z (PMC8569198; doi:10.1038/s41598-021-01163-z)
Supplement: Supplementary file 3 — Supplementary Information 3. [file 41598_2021_1163_MOESM3_ESM.docx]

**Supplementary Material 3. Average inlet and outlet concentration of N-NH_4_^+^. N-NO_3_^-^, and P-PO_4_^3-^ in the different experimental runs**

|  | **N-NH_4_^+^ (mg·L^-1^)^b^** | | **N-NO_3_^-^ (mg·L^-1^)^b^** | | **P-PO_4_^3-^ (mg·L^-1^)^b^** | |
| --- | --- | --- | --- | --- | --- | --- |
| **Run^a^** | **Inlet** | **Outlet** | **Inlet** | **Outlet** | **Inlet** | **Outlet** |
| **DM1 - WINTER** | | | | | | |
| **1** | 79.3 ± 2.4 | 0.3 ± 0.2 | 5.6 ± 0.6 | 19.5 ± 0.9 | 10.6 ± 1.4 | 4.1 ± 0.2 |
| **2** | 78.3 ± 3.6 | 2.4 ± 0.2 | 4.8 ± 0.5 | 9.7 ± 0.7 | 7.8 ± 1.1 | 2.4 ± 0.1 |
| **3** | 84.1 ± 3.9 | 0.4 ± 0.2 | 3.5 ± 0.4 | 12.6 ± 1.1 | 6.5 ± 1.2 | 1.8 ± 0.1 |
| **4** | 98.3 ± 4.2 | 3.7 ± 0.3 | 5.7 ± 0.4 | 16.7 ± 0.7 | 8.2 ± 0.5 | 1.9 ± 0.2 |
| **5** | 88.3 ± 4.1 | 0.0 ± 0.1 | 11.7 ± 0.6 | 2.9 ± 0.5 | 10.2 ± 1.8 | 1.6 ± 0.3 |
| **6** | 144.3 ± 6.2 | 0.3 ± 0.1 | 6.1 ± 0.6 | 1.3 ± 0.5 | 26.3 ± 2.2 | 2.1 ± 0.4 |
| **7** | 137.8 ± 9.3 | 0.1 ± 0.1 | 7.2 ± 0.3 | 1.9 ± 0.0 | 13.1 ± 1.3 | 3.0 ± 0.3 |
| **8** | 55.1 ± 4.1 | 8.4 ± 0.6 | 4.8 ± 0.2 | 15.7 ± 0.8 | 5.4 ± 0.6 | 1.9 ± 0.2 |
| **9** | 72.6 ± 5.1 | 4.8 ± 0.5 | 5.3 ± 0.4 | 21.9 ± 1.6 | 7.5 ± 0.4 | 3.2 ± 0.2 |
| **10** | 104.8 ± 3.0 | 0.5 ± 0.3 | 4.5 ± 0.5 | 1.8 ± 0.7 | 13.9 ± 0.2 | 1.2 ± 0.1 |
| **11** | 43.3 ± 2.1 | 0.4 ± 0.2 | 7.6 ± 0.2 | 15.1 ± 1.3 | 4.1 ± 0.0 | 2.9 ± 0.6 |
| **DM2 - SPRING** | | | | | | |
| **1** | 125.8 ± 6.2 | 5.8 ± 0.5 | 6.1 ± 1.0 | 23.0 ± 1.1 | 9.6 ± 0.3 | 0.2 ± 0.1 |
| **2** | 92.3 ± 5.2 | 0.3 ± 0.2 | 7.2 ± 1.1 | 6.4 ± 0.6 | 17.2 ± 1.9 | 6.4 ± 0.2 |
| **3** | 113.6 ± 9.3 | 0.0 ± 0.1 | 4.8 ± 1.0 | 5.9 ± 0.2 | 11.3 ± 1.6 | 1.5 ± 0.2 |
| **4** | 103.3 ± 5.3 | 1.0 ± 0.2 | 5.3 ± 1.2 | 16.7 ± 1.4 | 10.0 ± 1.1 | 2.3 ± 0.4 |
| **5** | 105.5 ± 2.1 | 6.3 ± 0.1 | 4.5 ± 0.6 | 13.1 ± 1.6 | 15.7 ± 0.9 | 3.6 ± 0.3 |
| **6** | 131.4 ± 2.3 | 8.4 ± 0.3 | 7.6 ± 0.4 | 1.9 ± 0.2 | 23.4 ± 3.6 | 4.5 ± 1.1 |
| **7** | 114.8 ± 3.5 | 0.7 ± 0.1 | 5.3 ± 0.3 | 4.9 ± 0.3 | 19.3 ± 4.2 | 2.1 ± 0.3 |
| **8** | 65.2 ± 3.1 | 2.1 ± 0.1 | 2.7 ± 0.2 | 23.7 ± 1.3 | 6.9 ± 1.5 | 1.8 ± 0.7 |
| **9** | 78.5 ± 3.6 | 0.7 ± 0.2 | 2.0 ± 0.2 | 20.3 ± 1.7 | 11.4 ± 3.6 | 4.6 ± 0.3 |
| **10** | 125.4 ± 4.1 | 9.3 ± 0.2 | 2.7 ± 0.6 | 3.9 ± 0.6 | 23.4 ± 2.9 | 0.3 ± 0.1 |
| **11** | 54.8 ± 3.0 | 1.9 ± 0.1 | 3.1 ± 0.8 | 4.8 ± 0.2 | 4.6 ± 0.2 | 1.8 ± 0.4 |
| **DM3 - SUMMER** | | | | | | |
| **1** | 105.8 ± 9.1 | 0.6 ± 0.1 | 2.4 ± 0.3 | 15.1 ± 1.5 | 14.3 ± 4.5 | 3.0 ± 0.8 |
| **2** | 88.3 ± 6.2 | 0.2 ± 0.2 | 6.1 ± 0.2 | 16.2 ± 2.3 | 15.3 ± 3.6 | 1.9 ± 0.3 |
| **3** | 103.6 ± 7.2 | 1.0 ± 0.3 | 7.2 ± 0.9 | 2.0 ± 0.7 | 14.0 ± 7.0 | 3.2 ± 0.2 |
| **4** | 103.3 ± 3.7 | 0.3 ± 0.1 | 4.8 ± 0.8 | 9.6 ± 0.8 | 7.1 ± 2.1 | 1.2 ± 0.2 |
| **5** | 105.5 ± 6.8 | 5.5 ± 0.8 | 5.3 ± 0.2 | 21.5 ± 3.6 | 14.6 ± 1.1 | 2.5 ± 0.5 |
| **6** | 121.4 ± 3.9 | 0.6 ± 0.6 | 4.5 ± 0.7 | 7.4 ± 2.9 | 25.1 ± 5.9 | 6.4 ± 0.2 |
| **7** | 114.8 ± 2.5 | 0.6 ± 0.2 | 7.6 ± 0.3 | 3.9 ± 0.5 | 29.7 ± 6.8 | 1.5 ± 0.1 |
| **8** | 75.2 ± 1.5 | 2.4 ± 0.7 | 5.3 ± 0.4 | 19.7 ± 5.3 | 9.2 ± 2.1 | 2.3 ± 0.1 |
| **9** | 68.5 ± 1.0 | 0.2 ± 0.0 | 2.7 ± 0.2 | 11.8 ± 0.4 | 13.7 ± 3.5 | 3.6 ± 0.4 |
| **10** | 134.4 ± 2.1 | 0.3 ± 0.1 | 2.0 ± 0.1 | 9.3 ± 2.5 | 22.7 ± 3.7 | 0.5 ± 0.1 |
| **11** | 54.8 ± 6.3 | 1.5 ± 0.1 | 3.2 ± 0.1 | 1.7 ± 0.7 | 2.9 ± 0.9 | 0.6 ± 0.0 |
| **DM4 – AUTUMN** | | | | | | |
| **1** | 132.0 ± 6.2 | 0.4 ± 0.2 | 7.2 ± 0.2 | 9.7 ± 0.6 | 11.2 ± 1.8 | 3.2 ± 0.1 |
| **2** | 72.3 ± 3.9 | 0.4 ± 0.1 | 4.8 ± 0.8 | 2.9 ± 0.5 | 13.1 ± 2.9 | 3.0 ± 1.6 |
| **3** | 105.6 ± 1.4 | 0.3 ± 0.1 | 5.3 ± 0.6 | 6.8 ± 0.7 | 8.2 ± 0.7 | 1.9 ± 0.5 |
| **4** | 85.6 ± 2.5 | 0.6 ± 0.3 | 4.5 ± 0.3 | 7.4 ± 0.1 | 14.0 ± 6.3 | 3.2 ± 0.9 |
| **5** | 112.5 ± 3.7 | 1.1 ± 0.4 | 7.6 ± 0.4 | 8.6 ± 0.7 | 16.0 ± 5.5 | 1.2 ± 0.7 |
| **6** | 125.8 ± 3.3 | 2.7 ± 0.1 | 7.3 ± 0.4 | 2.6 ± 0.4 | 29.4 ± 5.9 | 0.5 ± 0.1 |
| **7** | 101.5 ± 6.2 | 1.9 ± 0.6 | 4.5 ± 0.6 | 9.9 ± 0.2 | 13.4 ± 0.8 | 1.5 ± 0.7 |
| **8** | 83.6 ± 7.1 | 0.7 ± 0.2 | 7.6 ± 0.5 | 16.8 ± 7.2 | 5.3 ± 0.7 | 1.5 ± 0.1 |
| **9** | 83.3 ± 5.1 | 0.2 ± 0.0 | 4.8 ± 0.3 | 4.7 ± 0.8 | 6.5 ± 1.0 | 2.3 ± 0.6 |
| **10** | 115.5 ± 6.9 | 2.8 ± 0.1 | 3.5 ± 0.1 | 11.6 ± 2.2 | 25.4 ± 7.3 | 2.1 ± 0.4 |
| **11** | 61.1 ± 4.2 | 1.9 ± 0.3 | 1.2 ± 0.2 | 9.7 ± 0.3 | 3.9 ± 1.1 | 1.5 ± 0.2 |

^a^ Runs are described in Table 1.

^b^ Values represent the average of two experiments ± S.D. Determinations were conducted in triplicate.
